# Primary Distal Ileal Volvulus Presenting As Acute Intestinal Obstruction in an Adult: A Case Report

**DOI:** 10.7759/cureus.113333

**Published:** 2026-07-24

**Authors:** Akash Panda, Sourav Chakraborty, Shrimonti Sen, Udit K Das, Mala Mistri

**Affiliations:** 1 General Surgery, R. G. Kar Medical College and Hospital, Kolkata, IND; 2 Pathology, R. G. Kar Medical College and Hospital, Kolkata, IND

**Keywords:** acute abdomen, acute intestinal obstruction, double-barrel ileocolostomy, exploratory laparotomy, small bowel obstruction, small bowel volvulus, terminal ileal volvulus

## Abstract

Volvulus is defined as the abnormal twisting of a segment of the gastrointestinal tract around its mesenteric axis, resulting in luminal obstruction with potential compromise of the mesenteric blood supply. While colonic volvulus is common in adults, small-bowel volvulus is rare and represents a surgical emergency due to the high risk of bowel ischemia, necrosis, and sepsis. Although adult primary ileal volvulus has previously been described, isolated distal ileal volvulus without identifiable secondary pathology remains uncommon. A 52-year-old female with no comorbidities presented with a three-day history of progressively worsening abdominal pain, obstipation, and abdominal distension. Clinical examination revealed right iliac fossa tenderness with a palpable cystic swelling and features suggestive of small-bowel obstruction. Radiological evaluation demonstrated dilated small-bowel loops with air-fluid levels on plain radiography, and contrast-enhanced computed tomography revealed a transition point in the distal ileum with twisted bowel loops, consistent with ileal volvulus. Emergency exploratory laparotomy revealed a 360-degree anticlockwise volvulus of approximately 20 cm of terminal ileum with irreversible ischemic changes. Resection of the gangrenous ileal segment along with limited caecal resection was performed, followed by exteriorization as a double-barrel ileocolostomy. The patient had an uneventful recovery apart from a minor surgical site infection and subsequently underwent successful stoma reversal two months later. Histopathology confirmed transmural ischemic necrosis without evidence of malignancy or tuberculosis. Preoperative diagnosis remains difficult because clinical features are nonspecific, although contrast-enhanced CT can provide important clues when ileal volvulus is suspected. Isolated distal ileal volvulus remains an uncommon entity, and maintaining a high index of suspicion together with careful interpretation of CT findings may facilitate early diagnosis and improve outcomes.

## Introduction

Volvulus refers to abnormal twisting of a segment of the gastrointestinal tract around the axis of its mesentery, resulting in luminal obstruction and potential compromise of mesenteric blood supply. The term is derived from the Latin word volvere, meaning “to roll or twist.” The annual incidence of small-bowel volvulus is estimated to be 1.7-5.7 cases per 100,000 adults in Western countries, whereas considerably higher incidences of 24-60 cases per 100,000 have been reported in Asia, Africa, and the Middle East, including the Indian subcontinent. This geographical variation has been attributed to differences in dietary habits and meal patterns, although the exact pathophysiological mechanism remains incompletely understood [1].

Ileal volvulus, a subtype of small-bowel volvulus (SBV), is defined as torsion of a segment of ileum around its mesenteric axis. Although uncommon, it represents a surgical emergency due to rapid progression to bowel ischemia, necrosis, perforation, and sepsis. SBV should therefore be considered in patients presenting with acute abdominal pain and features of small-bowel obstruction [2].

Based on aetiology, SBV may be classified into neonatal, primary, and secondary types. Neonatal volvulus is commonly associated with intestinal malrotation and defective mesenteric fixation. Primary SBV occurs without identifiable pathology, and its mechanism remains unclear. Secondary SBV develops due to acquired conditions such as postoperative adhesions, congenital or acquired bands, mesenteric or omental defects, tumours, pregnancy, diverticular disease, or stomas [2,3].

Abdominal pain is the most common presenting symptom. The higher incidence in these regions has been attributed to dietary habits, particularly ingestion of large fibre-rich meals following prolonged fasting [4]. Although adult primary ileal volvulus has previously been described, isolated distal ileal volvulus without identifiable secondary pathology remains uncommon [5]. Diagnosis of SBV is challenging due to nonspecific clinical features and variable imaging findings. Delay in diagnosis and surgery may result in bowel necrosis, perforation, septic shock, multiorgan failure, and death, with reported mortality rates of 10%-38% depending on ischemia duration and timing of intervention [1,2].

## Case presentation

A 52-year-old female with no known comorbidities and post-menopausal status presented with a three-day history of abdominal pain. The pain was acute in onset, colicky in character, initially localized to the right iliac fossa, non-radiating, aggravated by oral intake, and partially relieved by rest. It gradually became generalized and intensified over the preceding 24 hours. She also reported non-passage of stool for two days and absence of flatus for one day. There was no history of fever, prior abdominal surgery, per-rectal or per-vaginal bleeding, weight loss, or similar episodes. The patient reported ingestion of a large fibre-rich meal following a period of religious fasting two days prior to onset of symptoms.

On examination, the patient appeared ill, febrile (100.2°F), and tachycardic (110 beats/min). Blood pressure and respiratory parameters were normal. Abdominal examination revealed distension and marked tenderness in the right iliac fossa with a soft ill-defined cystic mass. Bowel sounds were exaggerated. Digital rectal examination revealed a collapsed rectum without stool staining. Other systemic examinations were unremarkable.

**Figure 1:**
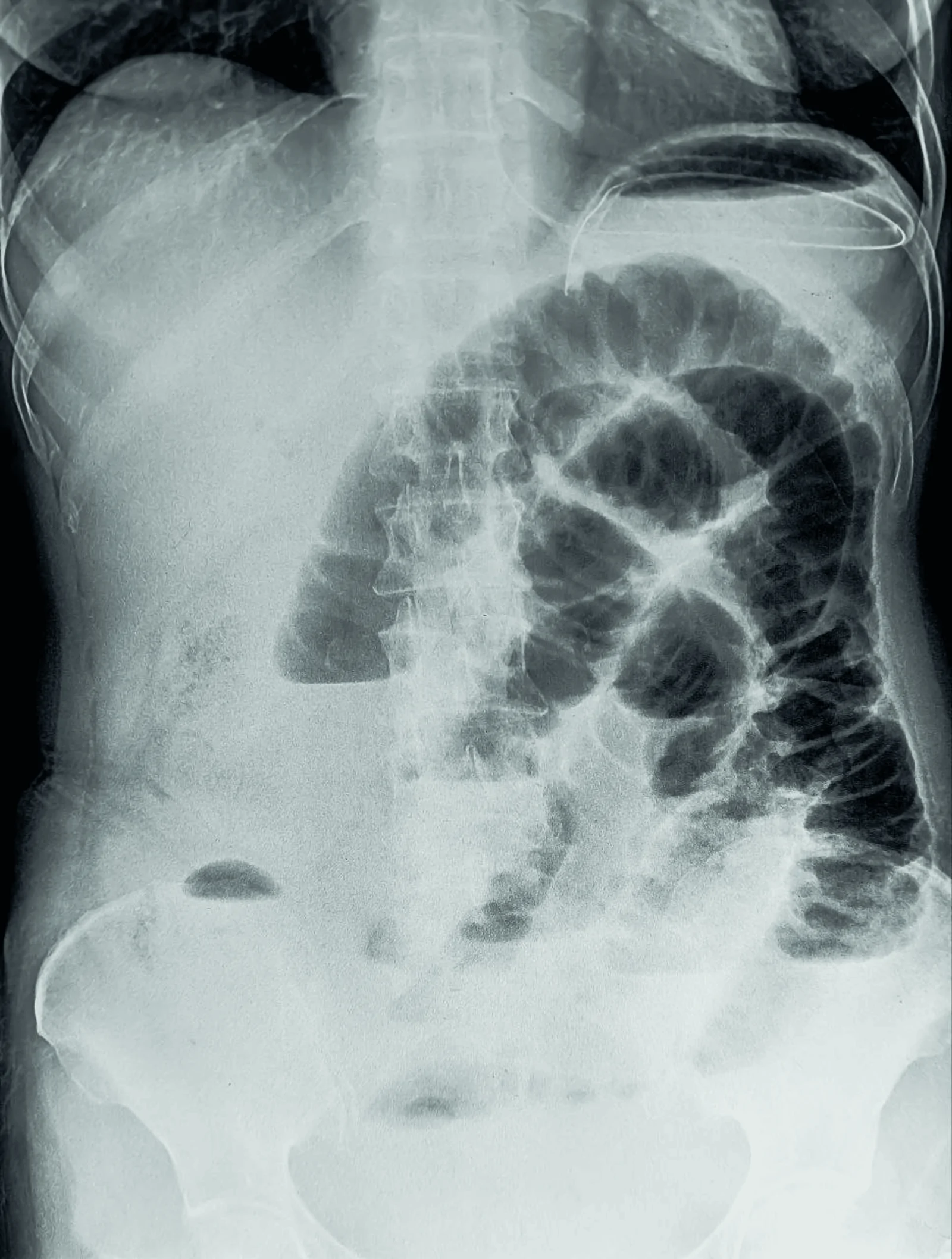
Digital straight X-ray of abdomen in erect posture consistent with the features of small-bowel obstruction

Laboratory investigations revealed haemoglobin of 9.6 g/dL, leucocytosis (total leukocyte count: 13.6 × 10⁹/L), hyponatremia (serum sodium: 128 mmol/L), serum potassium of 4.8 mmol/L, blood urea of 56 mg/dL, serum creatinine of 1.4 mg/dL, and C-reactive protein of 66 mg/L. Arterial blood gas analysis demonstrated a pH of 7.34 with a serum lactate level of 2.6 mmol/L, suggesting early tissue hypoperfusion without significant metabolic acidosis. Plain erect abdominal radiography demonstrated multiple air-fluid levels with dilated small-bowel loops and visible valvulae conniventes, with absence of distal bowel gas, consistent with small-bowel obstruction (SBO) (Figure 1).

**Figure 2:**
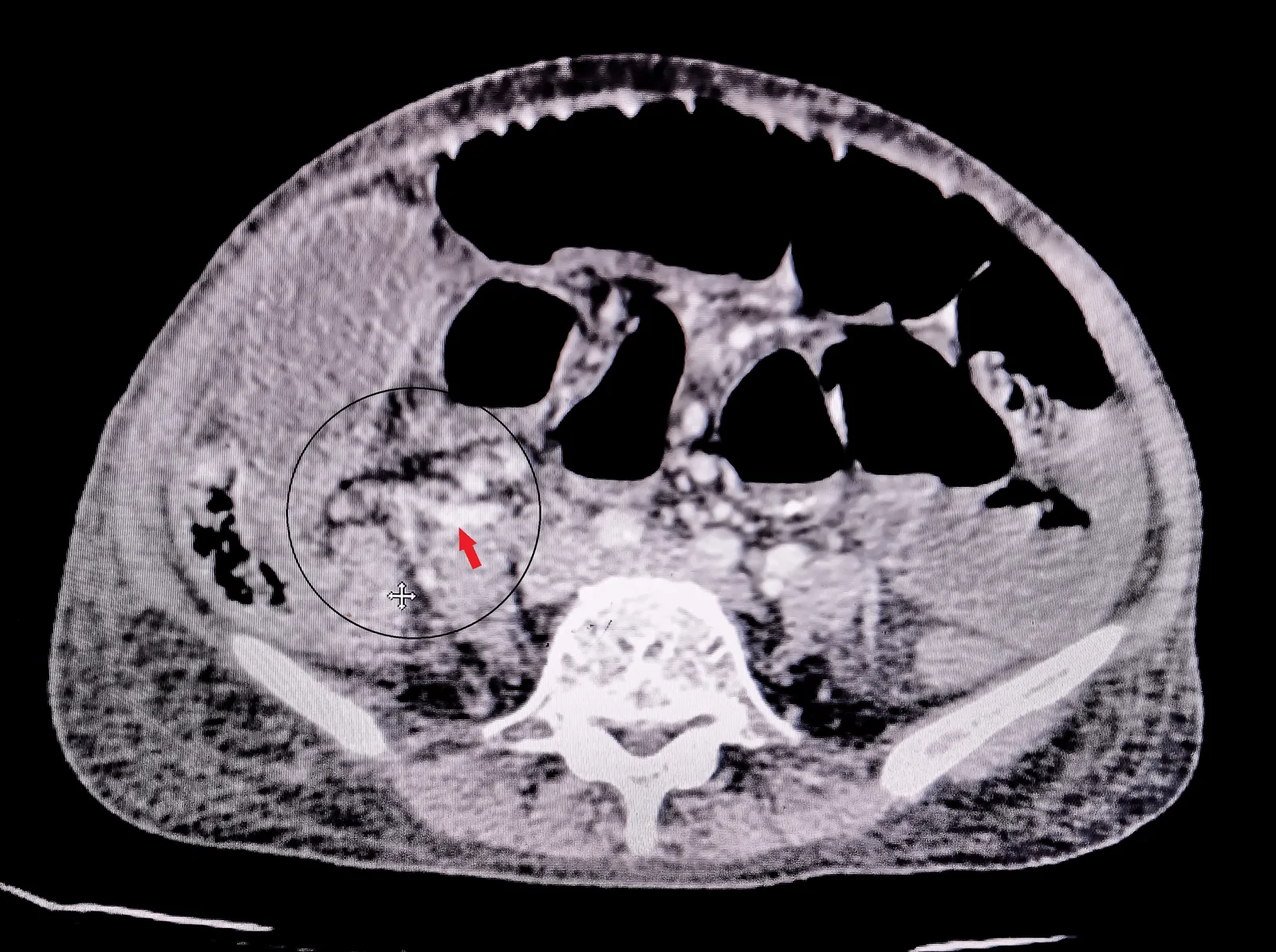
Contrast-enhanced computed tomography (CECT) whole abdomen showing twisted and conglomerated distal ileum and its twisted vasculature Distal ileum and its twisted vasculature (encircled); twisted vessels in the mesentery (marked with red arrow).

Contrast-enhanced computed tomography (CECT) of abdomen showed dilated distal jejunal and proximal ileal loops with air-fluid levels and hypodense trapped air producing a string-of-pearls appearance. An abrupt transition point was noted in the distal ileum in the right iliac fossa. The terminal ileum appeared twisted and conglomerated, with twisted vessels in the mesentery and a collapsed cecum and colon, consistent with ileal volvulus (Figure 2).

**Figure 3:**
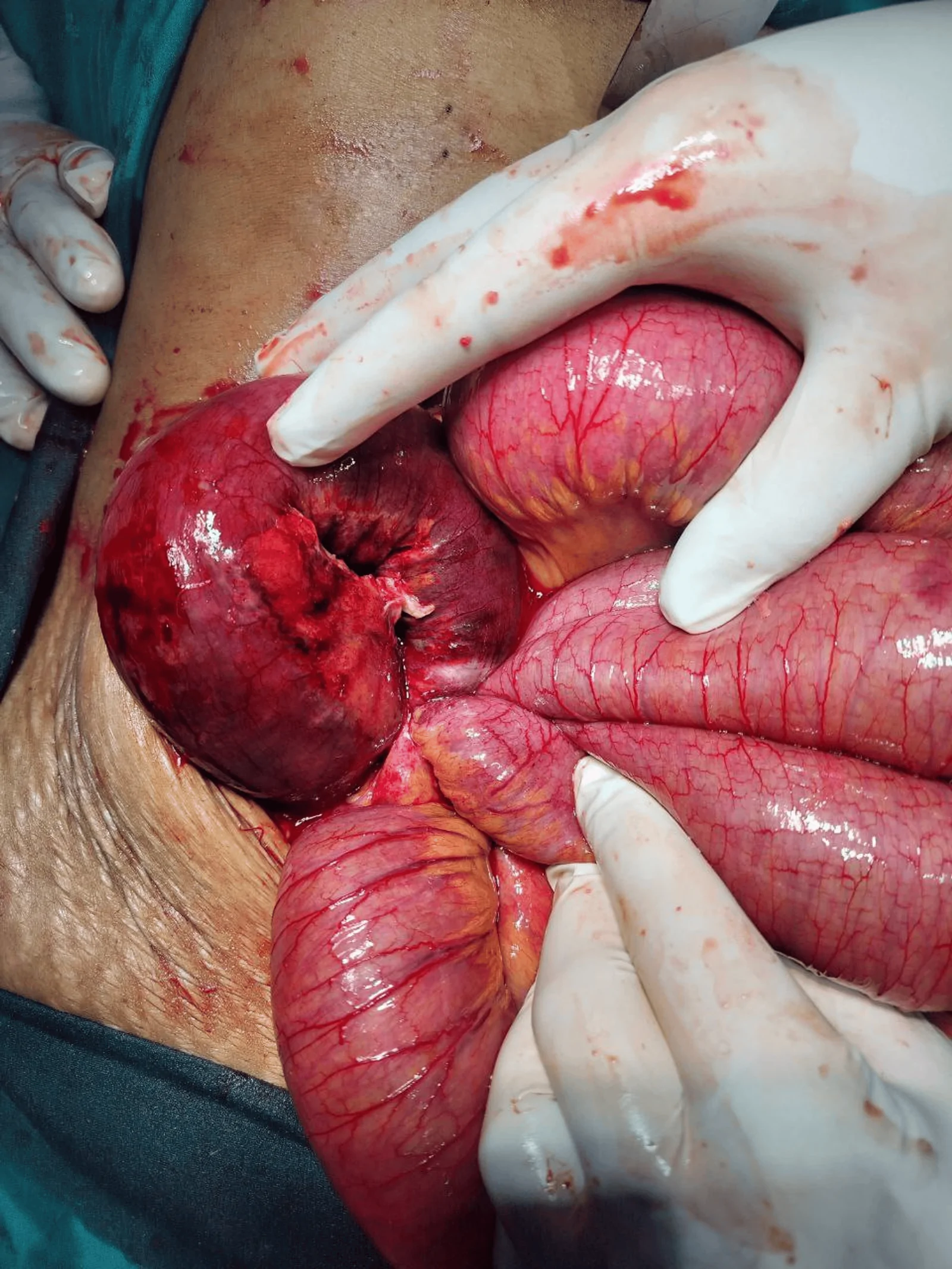
Distal ileal volvulus showing a dusky, congested, and non-viable bowel segment.

After resuscitation, exploratory laparotomy was performed after taking written informed consent. A small amount of serous ascitic fluid was present. The small bowel was grossly distended with a 360-degree anticlockwise twist involving approximately 20 cm of terminal ileum and its mesentery. The cecum and colon were collapsed (Figure 3).

**Figure 4:**
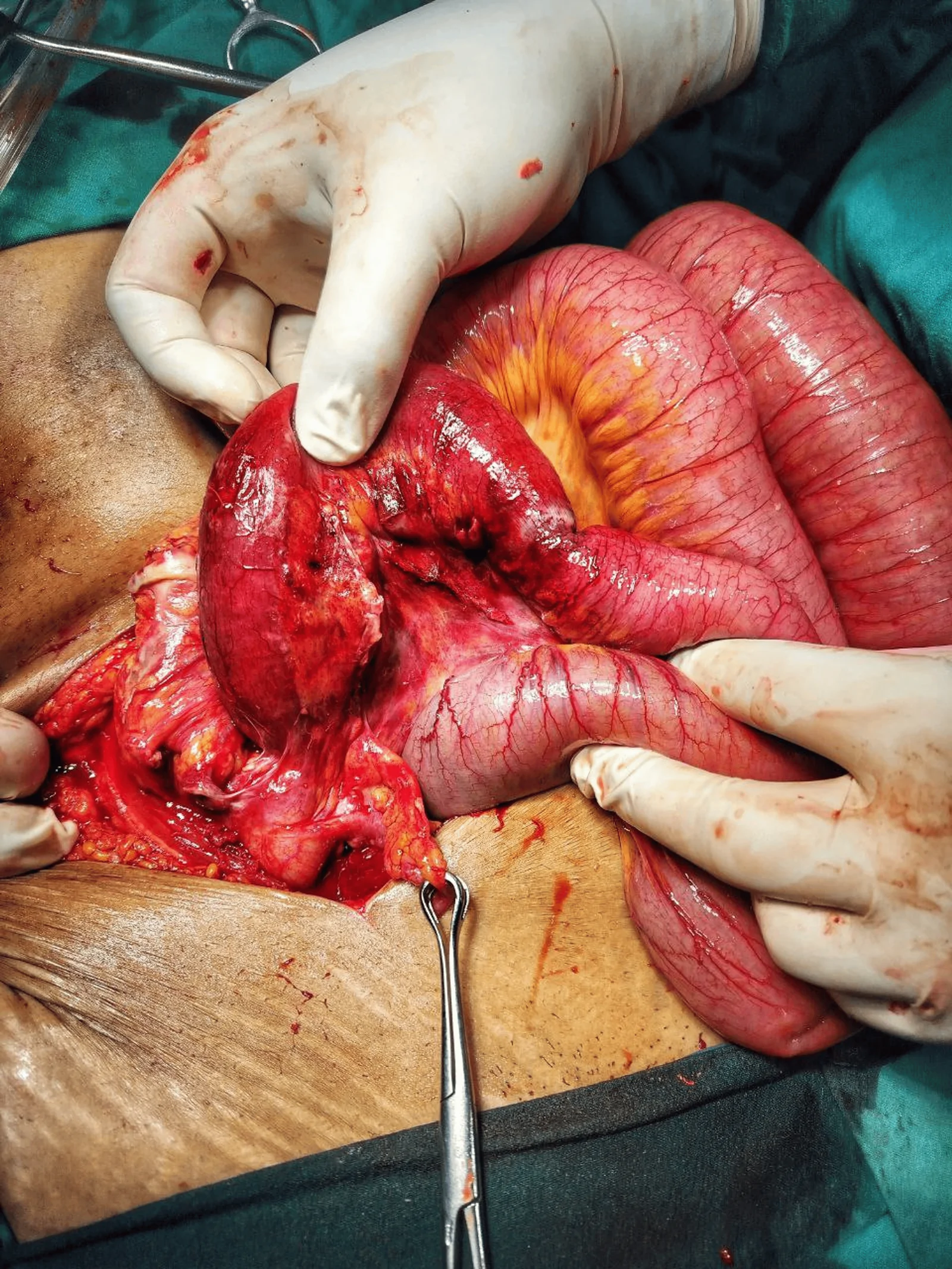
Distal ileum after manual derotation of the volvulus, the segment remained dusky, non-viable, aperistaltic Appendix (held with a pair of Babcock’s forceps).

The involved ileal segment was markedly congested, oedematous, dusky, and aperistaltic, with loss of normal bowel sheen, suggestive of irreversible ischemia. Mesenteric oedema was present, and arterial pulsations within the affected mesentery were markedly diminished. The proximal small bowel was markedly dilated with fluid-filled intestinal contents, while the distal bowel and colon remained collapsed beyond the transition point. Following gentle derotation and a period of warm saline packing with 100% oxygen ventilation, no improvement in bowel colour, peristalsis, or mesenteric pulsations was observed, confirming non-viability (Figure 4). Unfortunately, adjunctive methods for assessment of bowel viability, such as indocyanine green (ICG) fluorescence imaging, were unavailable in our setup.

Due to irreversible ischemia, resection of the distal 25 cm of terminal ileum along with 5 cm of cecum and appendix was performed. Because the patient presented after three days of obstruction with gross bowel oedema, questionable bowel viability following derotation, and a contaminated operative field after resection of gangrenous bowel, primary anastomosis was considered high risk. Therefore, a double-barrel ileocolostomy was performed to minimize the risk of anastomotic leakage, and a 28-French pelvic drain was placed.

**Figure 5:**
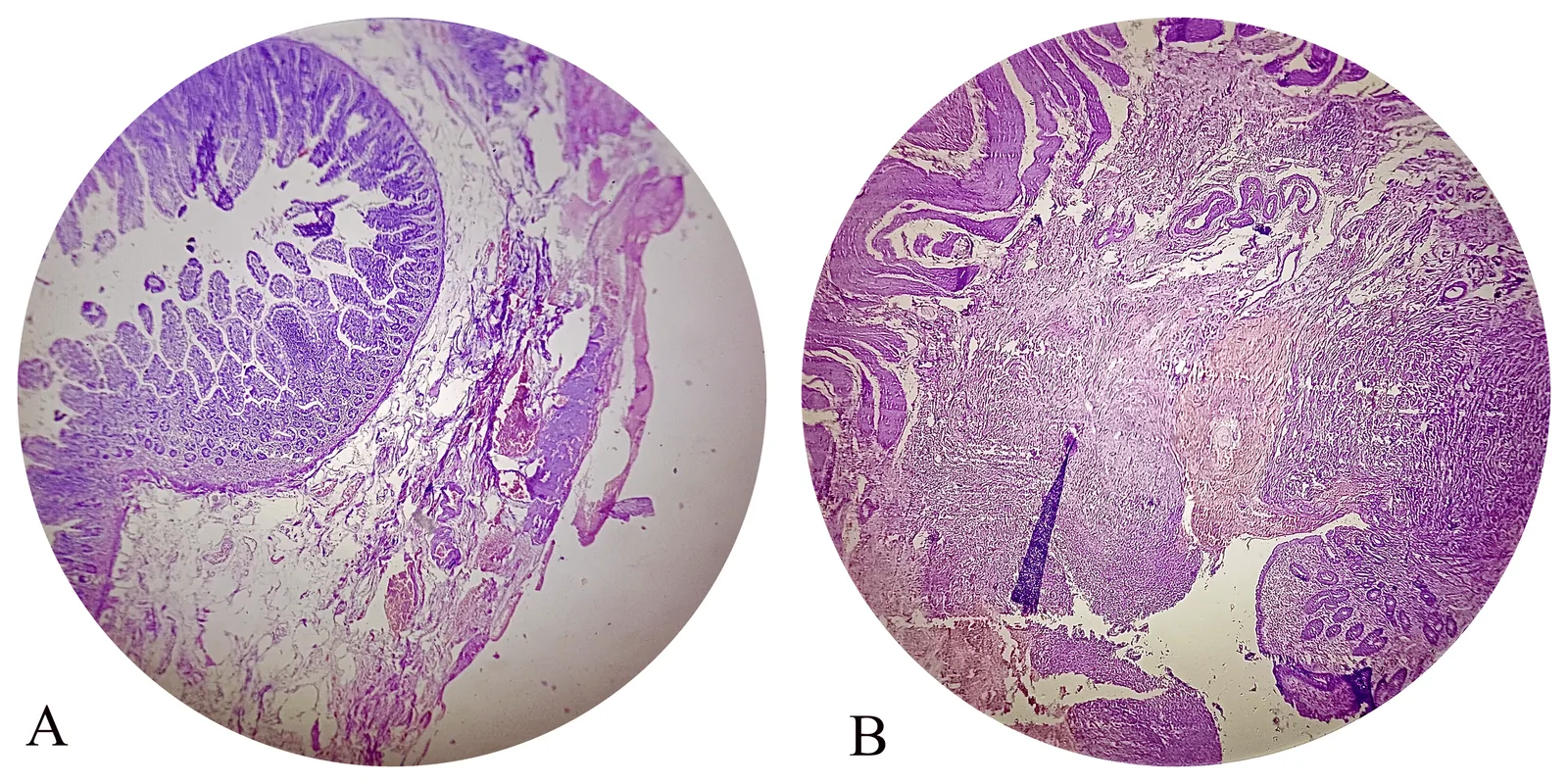
Haematoxylin and eosin (H&E)-stained section of the small intestine (A) Low-power view (x10) showing the small intestinal wall with villi and crypt (gland) formation. (B) Medium-power view (x40) demonstrating marked vascular congestion, oedema, and inflammatory cell infiltration throughout the intestinal wall.

The postoperative course was complicated by mild surgical site infection (SSI), managed conservatively. The patient was started on oral diet on postoperative day 2 when stoma became functional and was discharged on postoperative day 10. Histopathology showed acute inflammation with haemorrhage and transmural necrosis, without granuloma or malignancy (Figures 5A, 5B). Molecular testing for tuberculosis was negative.

Two months later, elective stoma reversal was performed. Proximal and distal bowel near the stoma were found to be congested and oedematous, and hence, approximately 5 cm of proximal and distal bowel was resected to obtain well-vascularized margins, and a tension-free side-to-side ileocolic anastomosis was done in four layers. The stoma site skin was left open for secondary closure. The patient was discharged on postoperative day 7, and follow-up was uneventful.

## Discussion

Primary ileal volvulus is an uncommon cause of acute intestinal obstruction in adults, particularly in patients without previous abdominal surgery or any identifiable secondary pathology [1,5]. Our patient presented with progressive intestinal obstruction, and CECT suggested distal ileal volvulus, which was confirmed during emergency laparotomy. Irreversible bowel ischemia necessitated bowel resection. Although the patient reported recent ingestion of a fibre-rich meal following religious fasting, this was considered only a temporal association rather than an established etiological factor [4].

The clinical presentation of primary SBV often overlaps with other causes of acute intestinal obstruction, making preoperative diagnosis challenging. Laboratory abnormalities such as leucocytosis, elevated inflammatory markers, electrolyte imbalance, and mild metabolic acidosis may indicate evolving bowel ischemia but are nonspecific. Contrast-enhanced CT remains the investigation of choice, with the whirl sign, twisting of mesenteric vessels, and an abrupt transition point being the most characteristic findings. In our patient, the CECT findings closely correlated with the operative diagnosis and facilitated timely surgical intervention before perforation or generalized peritonitis developed [2,3].

Management depends primarily on intraoperative assessment of bowel viability. When the bowel remains viable after derotation, simple devolvulation may be sufficient; however, gangrenous bowel requires resection. In our case, the involved bowel remained dusky, oedematous, and aperistaltic despite derotation, with no recovery of mesenteric pulsations, confirming irreversible ischemia. Owing to delayed presentation, gross bowel oedema, and the increased risk of anastomotic failure, a staged procedure with double-barrel ileocolostomy was preferred over primary anastomosis. Elective restoration of bowel continuity was subsequently performed with an uneventful recovery [2,6]. As primary ileal volvulus is uncommon in adults, we reviewed recently published cases of primary or isolated ileal volvulus to compare their clinical presentation, imaging findings, operative management, and outcomes with those of our patient (Table 1).

**Table 1:** Comparison of recently published adult cases of primary or distal ileal volvulus with the present case SBV: small-bowel volvulus, SBO: small-bowel obstruction, SSI: surgical site infection, US: ultrasound.

Author	Year	Age/Sex	Type	Predisposing factor	CT findings	Surgical procedure	Outcome	Reference
Bouassida et al.	2022	73/M	Primary SBV	None identified	CT suggestive of volvulus	Bowel resection with anastomosis	Uneventful recovery	[1]
Agrawal et al.	2020	60/M	Primary ileal volvulus	Fibre-rich meal after fasting	X-ray/US suggestive of SBO	Simple derotation	Uneventful recovery	[4]
Almaimani	2024	49/F	Primary ileal volvulus	None identified	Whirl sign on CT	Laparoscopic devolvulation	Uneventful recovery	[5]
El Nogoomi et al.	2024	19/M	Primary midgut volvulus	None identified	CT whirl sign	Resection	Uneventful recovery	[6]
Chand et al.	2025	52/F	Distal ileal volvulus	None identified	SBO with distal ileal volvulus	Resection + right hemicolectomy + ileocolostomy	Uneventful recovery	[7]
Faizan et al.	2020	50/F	Primary ileal volvulus	None identified	SBO features	Resection + double barrel ileostomy	Uneventful recovery	[8]
Present case	2026	52/F	Primary distal ileal volvulus	Possible temporal association with fibre-rich meal after fasting	CT, Twisted distal ileum with mesenteric vascular twisting and transition point	Resection + double-barrel ileocolostomy + delayed reversal	Uneventful recovery except mild SSI (managed conservatively)	

Despite variations in patient demographics and operative management, most published cases highlight the importance of early diagnosis and timely surgical intervention in preventing bowel loss and improving postoperative outcomes. Similar to our case, delayed presentation frequently resulted in bowel ischemia requiring resection, whereas patients diagnosed earlier could often be managed with simple devolvulation. These reports collectively emphasize that maintaining a high index of suspicion, carefully interpreting CT findings, and performing prompt surgical intervention are the key determinants of favourable outcomes [1,4-8].

## Conclusions

Primary distal ileal volvulus, although uncommon, should remain an important differential diagnosis in adults presenting with acute intestinal obstruction without previous abdominal surgery or identifiable secondary pathology. As demonstrated in our patient, early recognition, careful interpretation of CT findings, and prompt surgical intervention before irreversible bowel injury progresses are essential for achieving favourable outcomes. Individualized intraoperative decision-making, including staged reconstruction when bowel viability is compromised, can contribute to safe recovery and successful restoration of bowel continuity.
